# Construction of a Hantaan Virus Phage Antibody Library and Screening for Potential Neutralizing Activity

**DOI:** 10.3390/v15051034

**Published:** 2023-04-23

**Authors:** Zhuo Li, Huiyuan Zhang, Xiaxia Yu, Yusi Zhang, Lihua Chen

**Affiliations:** 1 Department of Immunology, The Fourth Military Medical University, 169 Changle West Road, Xi’an 710032, China; 2 Department of Medical Laboratory Technology, Xi’an Health School, Xi’an 710054, China; 3 Department of Immunology, Medicine School, Yan’an University, Yan’an 716000, China

**Keywords:** phage antibody library, Hantaan virus (HTNV), neutralizing antibodies (NAb)

## Abstract

China is one of the main epidemic areas for hemorrhagic fever with renal syndrome (HFRS). Currently, there is no human antibody specific to Hantaan virus (HTNV) for the emergency prevention and treatment of HFRS. To prepare human antibodies with neutralizing activity, we established an anti-HTNV phage antibody library using phage display technology by transforming peripheral blood mononuclear cells (PBMCs) of patients with HFRS into B lymphoblastoid cell lines (BLCLs) and extracting cDNA from BLCLs that secreted neutralizing antibodies. Based on the phage antibody library, we screened HTNV-specific Fab antibodies with neutralizing activities. Our study provides a potential way forward for the emergency prevention of HTNV and specific treatment of HFRS.

## 1. Introduction

Hemorrhagic fever with renal syndrome (HFRS) is an acute infectious disease caused by the Hantaan virus (HTNV) and is characterized by fever, hemorrhaging and acute renal failure [1,2,3]. Approximately 70–90% of cases occur in China, where infection is prevalent in most provinces and regions. The mortality rate is approximately 2–10% [4,5,6,7,8,9]. At present, there are only supportive and non-specific therapies against HTNV [10]. An anti-HTNV specific neutralizing antibody (NAb) could directly bind to HTNV and participate in immune clearance of the virus [11,12]. Although a murine monoclonal antibody (mAb) with HTNV-neutralizing activity was previously developed [13], the application of murine mAbs is limited due to their heterologous reactions [14,15]. Thus, the development of human mAbs for the emergency prophylaxis and treatment of HFRS is needed [1].

Phage surface display technology provides a way to prepare human mAbs [16,17,18]. Liang et al. applied the phage display technique and prepared a human Fab against HTNV using the lymphocytes from one convalescent patient with HFRS; however, this Fab bound only to HTNV and not to other types of hantavirus, so the library capacity was limited [19]. Koch et al. constructed an antibody library from the peripheral blood lymphocytes of four convalescent patients with HFRS and expressed and selected five recombinant IgG antibodies that showed neutralizing activity against HTNV and SEOV and, therefore, may be of value in the prevention and treatment of HFRS. The capacity of their library was approximately 10^6^ [20]. Therefore, finding novel methods to expand phage library capacity is of great significance.

In this study, to construct a phage library with a higher capacity, we collected peripheral blood mononuclear cells (PBMCs) from 35 people who were HTNV-Nab-positive HTNV vaccinated and patients with HFRS and transformed them into B lymphoblastoid cell lines (BLCLs) with the help of the Epstein–Barr virus (EBV). The cDNA was reverse transcribed based on the RNA extracted from the BLCLs [21]. The VH, VL, CH1 and CL domains of the Fab fragment were amplified, ligated and inserted via recombination into the phagocytic vector PHIAT-3 and then transfected into *E. coli* TG1. With the help of the helper phage, the phage antibody was packaged and synthesized, and a library of HTNV Fab phage antibodies with potential neutralizing activity was established. HTNV-specific Fab antibodies with neutralizing activities were subsequently screened out. Our study lays a foundation for obtaining neutralizing human antibodies against HTNV.

## 2. Materials and Methods

### 2.1. Materials, Antibodies and Cell Lines

*E. coli* TG1, helper phage M13K07, phage carrier pHIAT-3 and sheep anti-M13 antibody were purchased from Hongye Innovative Antibody Technologies Co., Ltd. (HIAT, Beijing, China). HTNV strain 76–118 was provided by the Department of Microbiology in the Fourth Military Medical University (Xi’an, China.). BLCLs were transformed and stored in our lab as described previously [22,23]. Briefly, the neutralizing-antibody-positive BLCLs were transformed from peripheral lymphocytes of patients with HFRS or vaccinated people immunized with HTNV using the EB virus, which was produced in the supernatant of B95-8 cells. The BLCLs were certified by detecting the surface expression of CD19 and HLA-DR. Taq polymerase, pfu polymerase, *HindIII* and *NotI* enzymes were purchased from TaKaRa. T4 DNA ligase was purchased from NEB Corporation. RNAiso Plus, PrimeScript™ II 1st Strand cDNA Synthesis Kit came from TaKaRa.

### 2.2. Construction of the Anti-HTNV Fab Phage Antibody Library

RNA was extracted from 1 × 10^8^ neutralizing-antibody-positive BLCLs [24] using RNAiso Plus (Takara). The first-strand cDNA was obtained with reverse transcription using the PrimeScript™ II 1st Strand cDNA Synthesis Kit from TaKaRa (Code No. 6210A). The absorbances at 260 and 280 nm were read using an ultraviolet spectrophotometer. The formula for the calculation of the RNA concentration was A260 × number of dilutions × 40 g/mL. The formula for the calculation of the DNA concentration was A260 × number of dilutions × 50 g/mL. The purity of the RNA or DNA was assessed based on the A260/A280 ratio.

The process for Fab phage antibody library construction is summarized in Figure S1. The primers were designed and synthesized by HIAT (Table S1). The variable regions containing VH and VL were amplified from cDNA. The VH, VL, CH1 and CL domains were linked to the Fab genes with PCR. Each step was confirmed by performing electrophoresis using 1% agarose gels. The PCR products were recovered and purified. *HindIII* and *NotI* were used to digest the PHIAT-3 bacteriophage vector and the Fab gene, respectively. T4 DNA ligase was used to insert the Fab gene into the PHIAT-3 bacteriophage vector. The resulting construct was then further electro-transformed into *E. coli* TG1. The transformed bacteria were serially diluted and then inoculated onto LBAG agar plates. The bacterial colonies were counted, and the original storage capacity was determined. The transformed TG1 colonies were randomly selected. Colony PCR was conducted to confirm whether the Fab gene was inserted into the phage DNA with the primers listed as follows:

Forward primer: L1: 5′-TggAATTgTgAgCggATAACAATT-3′

Reverse primer: S6: 5′-gTAAATgAATTTTCTgTATgAgg-3′

The PCR products were subjected to 1% agarose gel electrophoresis.

Then, 40 clones were randomly selected and sent to Sangon Biotech (Shanghai, China) Co., Ltd. for sequencing. The primers (HIAT) used for sequencing were as follows:

Forward primer: L1: 5′-TggAATTgTgAgCggATAACAATT-3′

Reverse primer: S6: 5′-gTAAATgAATTTTCTgTATgAgg-3′

### 2.3. Enrichment and Screening of the Anti-HTNV Fab Phage Antibody Library

The helper phage M13K07 was added to the above bacteria–phage antibody library with a ratio of phage/bacteria = 20:1. The phage antibody library was prepared after superinfection by resuspending the bacterial pellet with 200 μL 2 × YT-AK medium (containing both ampicillin and kanamycin) after centrifuging at 3500 rpm for 20 min and shaking overnight.

To prepare the initial phage antibody library, 1 mL of phage antibody library bacterial solution was diluted to OD600 = 0.3 with 2 × YT-AG medium (containing ampicillin and glucose) and cultured at 220 r/min in a shaker at 37 °C to OD600 = 0.5 (approximately 1 h). The helper phage M13K07 was added to the bacteria–phage antibody library with a ratio of phage/bacteria = 20:1 for infection and was gently shaken at 150 r/min at 37 °C for 1 h. After centrifugation at 5000 rpm at 4 °C for 10 min, the supernatant was discarded, and 50 mL of 2×YT-AK medium was added to resuspend the bacteria followed by shaking at 220 r/min overnight at 37 °C. The supernatant, containing an original Fab phage surface display library, was collected after centrifuging at 5000 rpm for 20 min at 4 °C.

A 96-well microplate was coated with the original 1 × 10^−7^ TCID/mL inactivated HTNV diluted 1:10 and blocked with PBS containing 3% BSA. To each well, 55 μL phage antibody library solution with a titer of 1.0 × 10^13^ PFU/mL was added with incubation at 37 °C for 2 h. The plate was then washed with PBS containing 0.1% Tween-20. During the first round of screening, washing was conducted 5 times; during the second round, washing was conducted 10 times; during the third round, washing was conducted 15 times with patting of excess liquid to dry. Distilled water was used to wash each well twice, and the wells were then left to absorb the liquid. To each well, 50 μL of glycine–hydrochloric acid elution buffer (pH = 2.2) was added followed by incubation at room temperature for 10 min. To neutralize the eluted phage solution, 3 μL of 2 M Tris was added to each well. The eluted phage solution was added to 2 mL newly cultured *E. coli* TG1 at A600 ≈ 1 and incubated at room temperature for 15–20 min. Then, 10 mL 2 × YT-A (containing ampicillin) culture medium was added to the bacterial solution. After mixing, 10 μL, 1 μL and 0.1 μL measures of bacterial solution were separately inoculated onto LB-A plates and then incubated at 37 °C overnight. The phage titers were calculated by counting clone numbers. The remaining bacterial solution was cultured with shaking at 37 °C for 1 h. Then, 100 mL 2 × YT-A was added, and the solution was cultured at 37 °C for another 1 h. Then, 1.0 × 10^12^ PFU phage M13K07 was added and cultured at 37 °C for 2 h. A total of 70 g/mL kanamycin was added and cultured overnight at 37 °C. The supernatant was collected in a tube and supplemented with 1/5 volume of PEG/NaCl. The tube was cooled in an ice bath for at least 1 h, and the pellet from centrifugation at 10,000 rpm for 20 min at 4 °C was then resuspended in 2 mL PBS. After filtration and sterilization, this was used for the next round of screening. The eluted bacteriophages after three or four rounds of enrichment screening were used for analysis and identification.

### 2.4. Preparation of the Monoclonal Phage-Ab

Fifty clones were randomly selected from the third and the fourth rounds of screening and were placed in 200 μL 2 × YT-AG medium and incubated with shaking overnight at 37 °C. The product was transferred to a new 200 μL measure of 2 × YT-AG medium at 1:100 and shaken for 2.5 h (OD600 ≈ 0.5) at 37 °C. A ratio of bacterial count/M13K07 = 1:20 was used for superinfection. The bacteria were centrifuged and resuspended with 200 μL 2 × YT-AK. After shaking overnight, the samples were centrifuged at 10,000 rpm for 15 min, and the supernatant was collected for identification.

### 2.5. Expression and Purification of the Fab Antibody

We placed 2 μL of phage culture supernatant of the positive clones in logarithmic growth into 400 μL of HB2151 culture solution phase and shook the solution at 150 rpm for 30 min at 37 °C. Then, the bacteria were inoculated onto LB-A plates and incubated at 30 °C overnight with TG1, HB2151 and recombinant phages used as controls. Next, a single colony was inoculated into 2 × YT-AG culture medium and shaken overnight at 250 rpm at 30 °C to OD600 nm ≈ 0.5–0.8. We then added 1.0 mmol/L IPTG to induce protein expression with overnight shaking at 250 rpm and incubation at 30 °C. The bacterial pellet was collected by centrifuging at 8000 rpm for 10 min at 4 °C, and the pellet was used to obtain antibodies in the periplasm with a final concentration of 0.5 mU of polymyxin B schizothrix. The bacterial periplasmic extracts were filtered with 0.45 μm membranes and stored at 4 °C or used for detection and purification.

The purification was performed according to the instructions of the HisPur Ni NTA kit (Smart-Lifesciences, Changzhou, China). After purification, the collected liquid was concentrated via ultrafiltration using a Millipore 10 KD ultrafiltration tube with centrifugation at 5000 rpm for 15 min at 4 °C.

### 2.6. Phage–ELISA Detection of the HTNV-Specific Fab Phage Antibodies

A microtiter plate was coated with 50 μL 1:100 diluted original TCID= 10^−7^/mL inactivated HTNV at 4 °C overnight. The wells were rinsed twice with deionized water. After blocking with 150 μL PBS containing 5% skimmed milk (dilution) per well at 37 °C for 1 h, 55 μL of 3× times diluted phage solution with a phage antibody titer of approximately 1.0 × 10^8^ PFU/mL was added to each well, and the plate was incubated at room temperature for l h. The plate was rinsed 10 times with PBST and dried. Then, 55 μL of mouse HRP-conjugated anti-phage M13 antibody diluted 1:1000 was added to each well. After incubation at 37 °C for 1 h, the plate was rinsed and developed using TMB substrate. The reaction was stopped by adding 2 M sulfuric acid. The plates were read at 450 nm. M13K07 Helper Phage was used as a negative control. The samples were defined as positive when positive/negative ≥ 2.1.

### 2.7. Microculture Neutralization Assay

The neutralization activities of the above-mentioned phage Fab antibodies were determined on Vero E6 cell monolayers in 96-well tissue culture plates. The Fab antibodies and controls were incubated with HTNV 76-118 strain (provided by the Department of Microbiology, Fourth Military Medical University) at 37 °C for 90 min. The 3D8 neutralization antibody was used as a positive control [25]. Virus–Fab antibody mixtures were applied to the cell monolayers, and the cells were incubated at 37 °C for 90 min in a CO_2_ incubator. Four replicate wells were prepared for each Fab antibody. Then, the culture medium with RPMI 1640 containing 2% FCS was replaced, and the cells were incubated for a further 14 days. The expression of HTNV nucleocapsid protein was detected using the 1A8 indirect ELISA kit as previously described [26]. Neutralizing activity was defined as the amount of antibody able to neutralize 50% of the virus.

### 2.8. Statistical Analysis

GraphPad Prism 5.0 Software was used for the statistical analysis of the experimental results. The Student’s *t*-test was used to compare the differences between the experimental group and the control group, and *p* < 0.05 indicated that the difference was statistically significant.

## 3. Results

### 3.1. Construction of the Anti-HTNV Fab Phage Antibody Library

**Figure 1:**
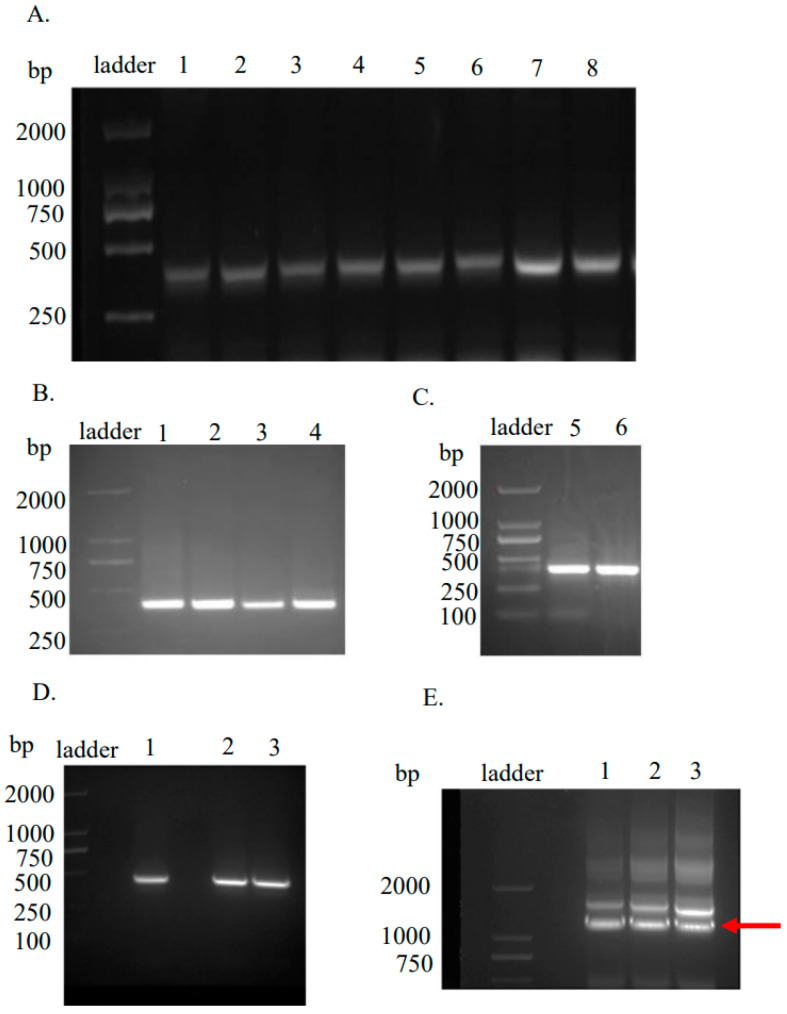
PCR amplification of the variable antibody regions with 1% agarose gel electrophoresis showing the PCR products of the VH (**A**), VL (**B**,**C**), CL (**D**, lane 1) and CH1 (**D**, lane 2 and lane 3) domains and the Fab fragment construct (**E, pointed by the red arrow**). The ladder is labeled in each gel image.

The RNA of the neutralizing-antibody-positive BLCLs (1 × 10^8^ cells) was extracted and reverse-transcribed into cDNA. The bands of approximately 350 bp for VH (Figure 1A), VL (Figure 1B,C), CH1 and CL (Figure 1D) were amplified and linked to the Fab gene fragment, confirmed by the appearance of the bands at approximately 1500 bp (lower band) (Figure 1E). The upper bands are byproducts resulting from homologous recombination.

### 3.2. Preparation and Characterization of the Anti-HTNV Fab Phage Antibody Library

**Figure 2:**
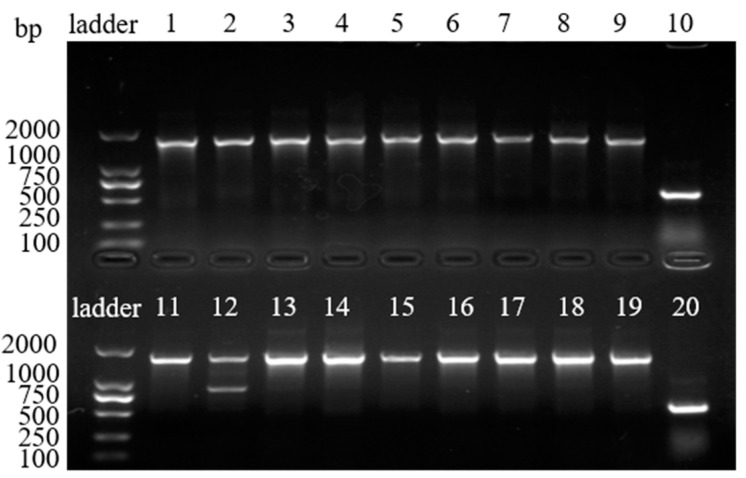
The amplified Fab products were identified using agarose gel electrophoresis. Lanes 1–9 and 11–19: random cloned PCR products. Lanes 10 and 20: negative controls.

Fab-pHIAT-3 was transformed into *E. coli* TG1. After screening with ampicillin, the bacterial clones were counted, and a gene bank of human Fab phage antibody with a reservoir capacity of 1.3 × 10^9^ was obtained. The Fab genes in 18 colonies were amplified using colony PCR; the amplified products were identified using agarose gel electrophoresis, which showed that the Fab fragment was successfully inserted into 17 clones, corresponding to an insertion rate of 94% (Figure 2). Although the Fab fragment was shown in lane 12, the byproduct was shown in this clone (Figure 2). Then, 40 clones were randomly selected and sequenced with the results of 27 clones of these indicating successful cloning. We defined the sequence accuracy by investigating whether the sequence had the insertion corresponding to the correct reading frame and found that the antibody sequence accuracy was 68%. After recovery of the recombinant phage by M13K07, a primary phage antibody library with a titer of 1.0 × 10^13^ PFU/mL was obtained.

### 3.3. Screening and Enrichment of the Antibody Library

The eluted phage titer of the primary phage antibody library was tested after four rounds of screening. The results show that the recovery rate increased step-by-step with the increasing number of screening rounds (Table 1), indicating that the positive clones were enriched after each round of screening.

**Figure 3:**
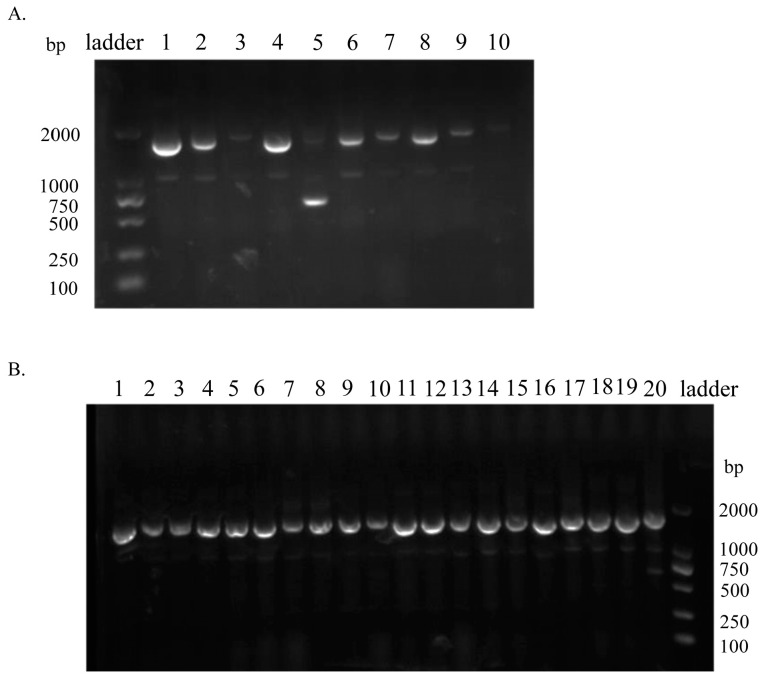
The identification of PCR products for the clones obtained after screening: Colony PCR identification for the (**A**; 1–10) first and (**B**; 1–20) second rounds of screening.

After the first and second rounds of screening, 10 and 20 clones, respectively, were randomly selected for colony PCR. The results show that after the first round of screening, there were seven clones with the Fab fragment (lanes 1, 2, 4, 6, 7, 8 and 9), and after the second round of screening, there were twenty clones with the Fab fragment, corresponding to insertion rates of 70% and 100%, respectively. These results suggested that, after screening, the positive clones were enriched (Figure 3).

We also compared the similarities of these sequences. We defined the Fab diversity by calculating the number of different sequences that account for the overall number of sequences. Before random screening, the accuracy of the clone sequencing was 68%, and the diversity was 95%. After four rounds of random screening, the accuracy of the clone sequencing was 81%, and the diversity was 31%.

### 3.4. Screening for Neutralizing Antibodies Specific to HTNV

**Figure 4:**
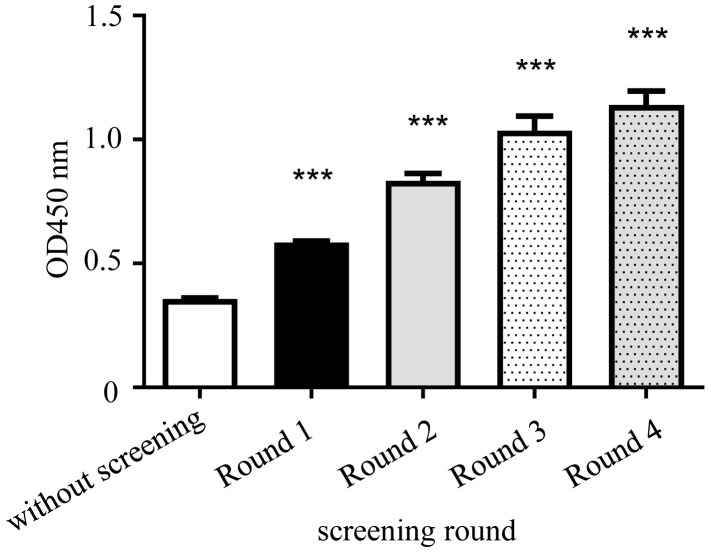
Phage ELISA results of the Fab phage antibody library. Compared with the original library (without screening), *** *p* < 0.001.

After four rounds of library screening for primary antibodies, phage ELISA using the bacteria liquid was used to inspect the saved phage antibody library after each round of screening. The results from the screening rounds one to four show that the OD value also gradually increased (Figure 4).

**Figure 5:**
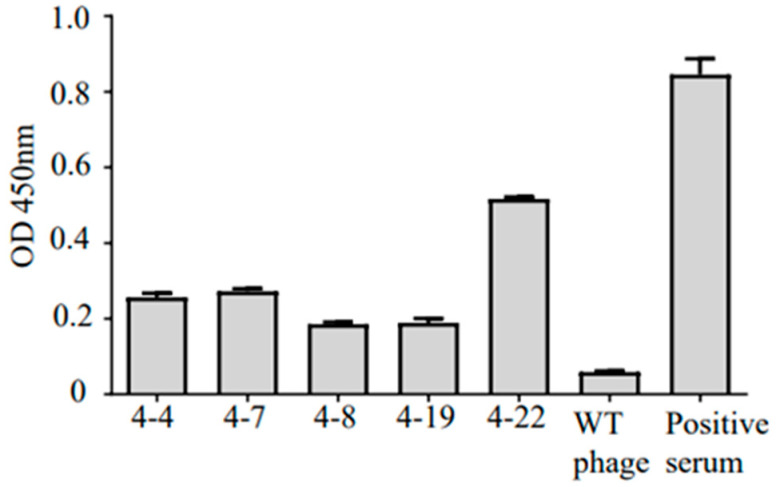
Phage ELISA was used to detect the combination of positive phage Ab and antigen. WT phage was used as a negative control, and serum from a patient with HFRS in the convalescent stage was used as a positive control.

To prepare the monoclonal phage antibody (phage-Ab), the phage antibodies eluted from each round were infected with TG1. The phage ELISA test results show that the positive rate gradually increased (Table 2). The positive rates for rounds three and four were similar. The results show that the positive clones were enriched after screening. Five Fab phage antibodies specific to HTNV were randomly selected from fifty clones during the third and the fourth rounds of screening (Figure 5).

Then, the phage antibodies were expressed and purified. A microculture neutralization assay was performed based on the above five HTNV-specific Fab antibodies following screening. As shown in Table 3, 5 μg of purified Fab antibody of clone 4–19 could protect over 50% Vero E6 cells from HTNV infection.

## 4. Discussion

Phage display technology is proven to be extremely practical for engineering antibody libraries. It provides a powerful tool for the high-throughput generation of affinity antibodies using an easy procedure. However, the antibody conformation may be altered during the panning process. Thus, constructing a phage library with larger capacity and diversity is necessary for the screening of specific antibodies, which largely depends on the quality of the constructed antibody library [27,28].

Previous studies constructed phage libraries directly from PBMCs of patients with HFRS. In our study, we enriched B cells from PBMCs by transforming PBMCs to BLCLs. Although EBV-transformed human BLCLs can secrete some human antibodies, the amount of secreted antibodies is small, and their quality is unstable, which creates difficulties in the preparation and acquisition of antibodies that result in limited clinical application prospects [22]. Unlike the natural phage antibody libraries, which are derived from unimmunized human or animal B lymphocytes, phage immune antibody libraries prepared from B lymphocytes of immunized humans or animals have no requirement for a large library capacity to obtain specific antibodies. Hence, we used BLCLs from 35 Nab-positive patients with HFRS and vaccinated people to construct the human immune antibody library and, thereby, ensure the diversity of antibody gene sources. During the construction of a phage antibody library, the capacity of the antibody library should exceed 10^14^ if all highly diverse VH and VL domains are randomly paired. However, it is reported that the capacity of antibody libraries is typically between 10^4^ and 10^9^ [29]. The next important step is to amplify as many antibody genes as possible to increase the library capacity and maintain diversity.

The amplified VH, VL, CL and CH1 domains were assembled into Fab fragments, which were cloned into the phage expression vector pHIAT 3 (Figure S2). *E. coli* TG1 was transformed via electroporation, and the number of clones was calculated to construct a human Fab phage antibody gene library with a library capacity of 1.3 × 10^9^. The primary phage antibody library had a titer of 1.0 × 10^13^ PFU/mL. The recombination rate is an important index used to measure the library capacity. Compared with the traditional identification method, colony PCR is simple and fast, and it is mainly used to identify the rate of gene recombination after transformation.

Phage antibodies can bind antigens and infect host bacteria for amplification. Phage surface display technology provides a convenient method for the rapid screening of specific antibodies [30]. Due to the absence of stable HTNV glycoprotein (GP), we used intact HTNV as an antigen to obtain phages with specific surface antibodies using the traditional solid phage screening method. Because the whole HTNV was directly used as an antigen, the effective concentration of envelope protein may have been very low and that of glycoprotein with neutralization epitopes even lower, which increased the difficulty of successful screening. These phage antibodies may have included both HTNV-NP- and HTNV-GP- specific antibodies. It is generally believed that Nab mainly targets HTNV-GP. HTNV-NP is also considered to be more immunogenic than HTNV-GP [19,31,32,33]. All these factors make it difficult to screen HTNV antibodies with neutralizing activity.

In addition, with the continuous expansion of the antibody library during the screening process, the growth rate of ineffective clones was much higher than that of bacteria expressing the antibody, resulting in the dilution of effective clones and increasing the difficulty of successful screening [34]. The cross-reaction of auxiliary phages and coated proteins affected the repeatability of the experiment and introduced inconvenience to the screening of specific antibodies using phage ELISA. Each step, including coating, blocking and washing, reduced the efficiency of screening. The results show that, after four rounds of screening, the recovery, positive rate and absorbance of the specific phage antibody were significantly increased, and the efficiency of the third round was similar to that of the fourth round. The recombinant rate of Fab reached 100% after the second round of screening. These results indicate that the HTNV-specific antibody was enriched. Finally, we screened five unique HTNV Fab phage antibodies from fifty clones in the third and fourth rounds of screening via sequencing analysis. These five phage Fab antibodies had different binding activities with HTNV.

There are limitations in this study. Since HTNV-GP has weak immunogenicity, and recombinant HTNV-GP antigen is not available for detection, these weakly reactive antibodies were also used for further analysis. To obtain antibodies with stronger affinity, more clones need to be selected for identification. In addition, we also need to identify the antigen (HTNV-GP or HTNV-NP) that the antibodies are targeting, which may directly affect their practical application. Finally, further studies on the affinity between antibody and HTNV antigen and structure are still needed.

## 5. Conclusions

In conclusion, we created a large and diverse Fab phage display library to enable the discovery of therapeutic HTNV antibodies. The antibody library lays a foundation for sequence analysis, expression and purification of neutralizing antibodies.

## Figures and Tables

**Table 1:** Recovery rates of phage antibodies during screening.

Rounds of Panning	Input (PFU)	Output (PFU)	Yield (%)
1st	1.0 × 10^12^	1.0 × 10^5^	1.0 × 10^−5^
2nd	1.0 × 10^9^	4.0 × 10^3^	4 × 10^−4^
3rd	1.0 × 10^8^	5.0 × 10^2^	5.0 × 10^−4^
4th	2.5 × 10^8^	6.0 × 10^2^	2.4 × 10^−4^

Note: yield (%) = (PFU of phage eluted) × 100/(PFU of phage applied).

**Table 2:** HTNV-specific antibody positive rate after screening.

Round	Positive Rate
Before panning	40%
Round 2	65%
Round 3	76%
Round 4	72%

**Table 3:** Neutralizing activity of the human Fab antibody against Hantaan virus.

Fab Antibody	Amount of Antibody Used to Reach TCID
4–4	−
4–7	−
4–8	−
4–19	+(5 μg)
4–22	−

“+” indicates positive: Fab antibodies that protected 50% of cells from infection with 100 TCID HTNV in the virus neutralization test. “−” indicates negative: Fab antibodies that did not prevent infection of 50% of cells with 100 TCID HTNV in the virus neutralization test.

## Data Availability

The authors declare that all data supporting the findings of this study are available within this article and its Supplementary Information files, or from the corresponding author upon reasonable request.

## Supplementary Materials

The following supporting information can be downloaded at: https://www.mdpi.com/article/10.3390/v15051034/s1, Figure S1: Schematic figure shows the construction process of the Fab phage antibody library; Figure S2: The pHIAT-3 plasmid map; Table S1: The primers of the constructed Fab phage antibody library.
