# Supplementary material for: Construction of a Hantaan Virus Phage Antibody Library and Screening for Potential Neutralizing Activity
Source: Viruses. 2023 Apr 23;15(5):1034. doi: 10.3390/v15051034 (PMC10221454; doi:10.3390/v15051034)
Supplement: Supplementary file 1 [file viruses-15-01034-s001.zip › viruses-2340561-supplementary.pdf]

Supplementary Figure 1. Schematic figure showed the construction process of Fab phage antibody library.

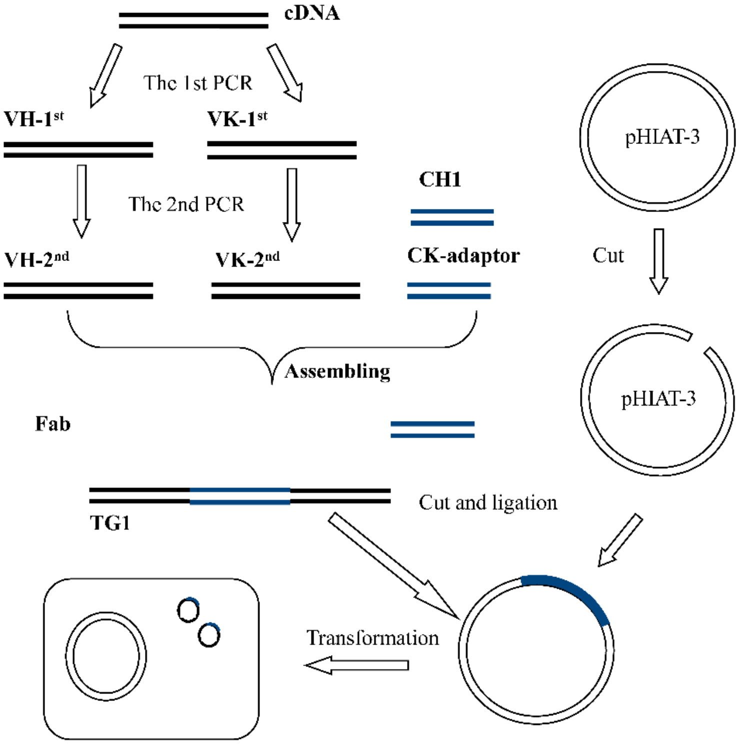

Supplementary Figure 2. The pHIAT-3 plasmid map.

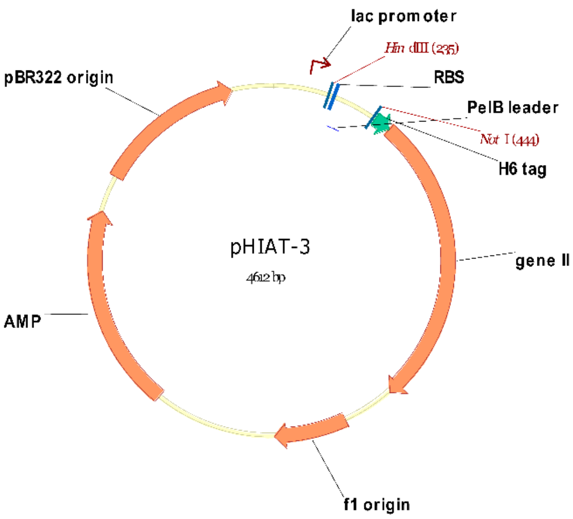

Table S1. The primers of constructed Fab phage antibody library

primer	sequence
VL1-1st-u-1	5’-GTTATTACTCGCAGCAAGCGGCGCGCATGCCGAAATTGTGCAGTCTGTSBTGACGCAGCCGCC-3’

VL1-1st-u-2	5’-GTTATTACTCGCAGCAAGCGGCGCGCATGCCGAAATTGTGTCCTATGWGCTGACWCAGCCAC-3’
VL1-1st-u-3	5’-GTTATTACTCGCAGCAAGCGGCGCGCATGCCGAAATTGTGTCCTATGAGCTGAYRCAGCYACC-3’
VL1-1st-u-4	5’-GTTATTACTCGCAGCAAGCGGCGCGCATGCCGAAATTGTGCAGCCTGTGCTGACTCARYC-3’
VL1-1st-u-5	5’-GTTATTACTCGCAGCAAGCGGCGCGCATGCCGAAATTGTGCAGDCTGTGGTGACYCAGGAGCC-3’
VL1-1st-u-6	5’-GTTATTACTCGCAGCAAGCGGCGCGCATGCCGAAATTGTGCAGCCWGKGCTGACTCAGCCMCC-3’
VL1-1st-u-7	5’-GTTATTACTCGCAGCAAGCGGCGCGCATGCCGAAATTGTGTCCTCTGAGCTGASTCAGGASCC-3’
VL1-1st-u-8	5’-GTTATTACTCGCAGCAAGCGGCGCGCATGCCGAAATTGTGCAGTCTGYYCTGAYTCAGCCT-3’
VL1-1st-u-9	5’-GTTATTACTCGCAGCAAGCGGCGCGCATGCCGAAATTGTGAATTTTATGCTGACTCAGCCCC-3’
VL1-1st-d	5’-CACCAGTGTGGCCTTGTTGGCTTG-3’
VL2-1st-u-1	5’-GTTATTACTCGCAGCAAGCGGCGCGCATGCCGAAATTGTGGACATCCRGDTGACCCAGTCTCC-3’
VL2-1st-u-2	5’-GTTATTACTCGCAGCAAGCGGCGCGCATGCCGAAATTGTGGAAATTGTRWTGACRCAGTCTCC-3’
VL2-1st-u-3	5’-GTTATTACTCGCAGCAAGCGGCGCGCATGCCGAAATTGTGGATATTGTGMTGACBCAGWCTCC-3’

VL2-1st-u-4	5'-GTTATTACTCGCAGCAAGCGGCGCGCATGCCGAAATTGTGGAAACGACACTCACGCAGTCTC-3'
VL2-1st-d	5'-GTTTCTCGTAGTCTGCTTTGCTCA-3'
VL1-2nd-u	5'-ATGAAATACCTATTGCCTACGGCAGCCGCTGGATTGTTATTACTCGCAGC-3'
VL1-2nd-d-1	5'-CAGCCTTTGGCTGTCCTAGGACGGTSASCTTGGTCC-3'
VL1-2nd-d-2	5'-CAGCCTTTGGCTGTCCTAGGACGGTCAGCTGGGTGC-3'
VL2-2nd-d-1	5'-GATGGCGCCGCCACCGTACGTTTGATTTCCACCTTGGTCC-3'
VL2-2nd-d-2	5'-GATGGCGCCGCCACCGTACGTTTGATCTCCASCTTGGTCC-3'
VL2-2nd-d-3	5'-GATGGCGCCGCCACCGTACGTTTGATATCCACTTTGGTCC-3'
VL2-2nd-d-4	5'-GATGGCGCCGCCACCGTACGTTTAATCTCCAGTCGTGTCC-3'
VH-1st-u-1	5'-CTGCCCAGCCGGCCATGGCCCAGGTGCAGCTGCAGGAGTCSG-3'
VH-1st-u-2	5'-CTGCCCAGCCGGCCATGGCCCAGGTACAGCTGCAGCAGTCA-3'
VH-1st-u-3	5'-CTGCCCAGCCGGCCATGGCCCAGGTGCAGCTACAGCAGTGGG-3'
VH-1st-u-4	5'-CTGCCCAGCCGGCCATGGCCGAGGTGCAGCTGKTGGAGWCY-3'
VH-1st-u-5	5'-CTGCCCAGCCGGCCATGGCCCAGGTCCAGCTKGTRCAGTCTGG-3'
VH-	5'-CTGCCCAGCCGGCCATGGCCCAGRTCACCTTGAAGGAGTCTG-3'

1st-u-6	
VH-	5’-CTGCCCAGCCGGCCATGGCCCAGGTGCAGCTGGTGSARTCTGG-3’
1st-u-7	
VH-	5’-CTGCCCAGCCGGCCATGGCCGAGGTGCAGCTGTTGGAGTCT-3’
1st-u-8	
VH-	5’-GGGAATTCTCAGAGGAGACGA-3’
1st-d-1	
VH-	5’-GGAAGGTGTGCACGCCGCTGGTC-3’
1st-d-2	
VH-	5’-TGCCCAGCCGGCCATGGCC-3’
2nd-u	
VH-	5’-CGATGGGCCCTTGGT GCTAGCTGAGGAGACRGTGACCAGGGTG-3’
2nd-d-1	
VH-	5’-CGATGGGCCCTTGGT GCTAGCTGAGGAGACGGTGACCAGGGTT-3’
2nd-d-2	
VH-	5’-CGATGGGCCCTTGGT GCTAGCTGAAGAGACGGTGACCATTGT-3’
2nd-d-3	
VH-	5’-CGATGGGCCCTTGGT GCTAGCTGAGGAGACGGTGACCGTGGTCC-3’
2nd-d-4	
CL2-u	5’-CGTACGGTGGCGGCGCCATC-3’
CL2-d	5’-GGCCATGGCCGGCTGGGCAG-3’
CL1-u	5’-ACCGTCCTAGGACAGCCAAAGGCTG-3’
CL1-d	5’-GGCCATGGCCGGCTGGGCAGCGAGT-3’
CH1-u	5’-GCTAGCACCAAGGGCCCATC-3’
CH1-d	5’-ACAGTAGCGGCCGCGTGGTGATGGTGATGATG-3’
Fab-u	5’-GCCAAGCTTGCCAAATTCTATTTCAAGGAGACAGTCATAATGAAATACCTATTGCCTAC-3’
Fab-d	5’-ACAGTAGCGGCCGCGTGGTGATGGTGATGATG-3’
